# *N*-Methyl-D-Aspartate Receptors in Hematopoietic Cells: What Have We Learned?

**DOI:** 10.3389/fphys.2020.00577

**Published:** 2020-06-17

**Authors:** Maggie L. Kalev-Zylinska, James I. Hearn, Asya Makhro, Anna Bogdanova

**Affiliations:** ^1^ Blood and Cancer Biology Laboratory, Department of Molecular Medicine and Pathology, University of Auckland, Auckland, New Zealand; ^2^ Department of Pathology and Laboratory Medicine, LabPlus Haematology, Auckland City Hospital, Auckland, New Zealand; ^3^ Red Blood Cell Research Group, Institute of Veterinary Physiology, Vetsuisse Faculty, University of Zurich, Zürich, Switzerland; ^4^ Zurich Center for Integrative Human Physiology, University of Zurich, Zürich, Switzerland

**Keywords:** glutamate, intracellular calcium signaling, megakaryocyte, erythropoiesis, red cells, platelets

## Abstract

The *N*-methyl-D-aspartate receptor (NMDAR) provides a pathway for glutamate-mediated inter-cellular communication, best known for its role in the brain but with multiple examples of functionality in non-neuronal cells. Data previously published by others and us provided *ex vivo* evidence that NMDARs regulate platelet and red blood cell (RBC) production. Here, we summarize what is known about these hematopoietic roles of the NMDAR. Types of NMDAR subunits expressed in megakaryocytes (platelet precursors) and erythroid cells are more commonly found in the developing rather than adult brain, suggesting trophic functions. Nevertheless, similar to their neuronal counterparts, hematopoietic NMDARs function as ion channels, and are permeable to calcium ions (Ca^2+^). Inhibitors that block open NMDAR (memantine and MK-801) interfere with megakaryocytic maturation and proplatelet formation in primary culture. The effect on proplatelet formation appears to involve Ca^2+^ influx-dependent regulation of the cytoskeletal remodeling. In contrast to normal megakaryocytes, NMDAR effects in leukemic Meg-01 cells are diverted away from differentiation to increase proliferation. NMDAR hypofunction triggers differentiation of Meg-01 cells with the bias toward erythropoiesis. The underlying mechanism involves changes in the intracellular Ca^2+^ homeostasis, cell stress pathways, and hematopoietic transcription factors that upon NMDAR inhibition shift from the predominance of megakaryocytic toward erythroid regulators. This ability of NMDAR to balance both megakaryocytic and erythroid cell fates suggests receptor involvement at the level of a bipotential megakaryocyte-erythroid progenitor. In human erythroid precursors and circulating RBCs, NMDAR regulates intracellular Ca^2+^ homeostasis. NMDAR activity supports survival of early proerythroblasts, and in mature RBCs NMDARs impact cellular hydration state, hemoglobin oxygen affinity, and nitric oxide synthase activity. Overexcitation of NMDAR in mature RBCs leads to Ca^2+^ overload, K^+^ loss, RBC dehydration, and oxidative stress, which may contribute to the pathogenesis of sickle cell disease. In summary, there is growing evidence that glutamate-NMDAR signaling regulates megakaryocytic and erythroid cells at different stages of maturation, with some intriguing differences emerging in NMDAR expression and function between normal and diseased cells. NMDAR signaling may provide new therapeutic opportunities in hematological disease, but *in vivo* applicability needs to be confirmed.

## Introduction

This review summarizes what has been learned about the roles of *N*-methyl-d-aspartate receptor (NMDAR) in megakaryocytic and erythroid cells. NMDARs are best known for their functions as glutamate-gated cation channels in the central nervous system (Traynelis et al., 2010). It appears that the NMDAR ion channel functionality is maintained in blood progenitors but NMDAR channel properties and its downstream pathways await further characterization in these cells. This paper starts with a brief overview of glutamate signaling in the brain. On this background, we highlight distinctive features of NMDAR in hematopoietic cells. Other glutamate receptors and mature blood cells are not discussed in detail but the appropriate background is provided to place this emerging field of research in a meaningful context. We describe NMDAR effects on hematopoietic differentiation, including some of our recent observations that suggest a novel role for the receptor in balancing megakaryocytic and erythroid cell fates (Hearn et al., 2020).

## Classical Glutamate-Nmdar Axis in the Brain

Glutamate is synthesized from glutamine as a part of normal cellular metabolism in all cells (Yelamanchi et al., 2016). In neurons, vesicular glutamate transporters (VGLUT) pump glutamate into pre-synaptic vesicles (Daikhin and Yudkoff, 2000; Zhou and Danbolt, 2014). Upon membrane depolarization, vesicles fuse with the pre-synaptic plasma membrane and glutamate is released into the synaptic cleft. This process engages soluble *N*-ethyl-maleimide-sensitive factor attachment protein receptor (SNARE) proteins that are activated by Ca^2+^ entry through voltage-gated Ca^2+^ channels. Following release, glutamate concentrations in the synaptic cleft increase markedly, from 2–5 μM to approximately 1.1 mM. While in the synaptic cleft, glutamate activates ionotropic and metabotropic receptors located on the post-synaptic plasma membrane (Reiner and Levitz, 2018). Ionotropic receptors function as ion channels (for Na^+^, K^+^, and Ca^2+^), and metabotropic receptors activate G-proteins that modulate ion channels directly and indirectly. The main purpose of the ionic flux is to generate and propagate action potentials characteristic of excitable tissues. The synaptic glutamate signal is terminated by the excitatory amino acid transporters (EAAT) present on astrocytes that remove glutamate from the synaptic cleft (Featherstone, 2010).

The family of ionotropic glutamate receptors includes NMDA, α-amino-3-hydroxy-5-methyl-4-isoxazolepropionic acid (AMPA), and kainate receptors, each named after a distinct, synthetic agonist that activates them (Traynelis et al., 2010). AMPA and kainate receptors respond to glutamate first. They mediate intracellular influx of mostly Na^+^, which leads to membrane depolarization and if large enough, triggers action potential. Membrane depolarization releases a Mg^2+^ ion blocking the pore of NMDAR, enabling receptor function. This order of events highlights that neuronal NMDAR can activate only when glutamate binding and membrane depolarization coincide (which is named “coincidence detection”). NMDAR-mediated Ca^2+^ influx contributes little to membrane depolarization but modifies synaptic strength through molecular events related to the Ca^2+^ role as “second messenger” (Traynelis et al., 2010; Hansen et al., 2018).

Typical NMDARs are built as tetramers that combine two obligate GluN1 subunits with another two GluN2 (A–D) or GluN3 (A or B) subunits, in various combinations. It is believed that GluN1 subunit is an essential component of all NMDARs, and variable GluN2 and GluN3 subunits are modulatory. NMDAR activation requires binding of l-glutamate on each of the GluN2 subunits, as well as glycine (co-agonist) on the GluN1 and GluN3 subunits. The alternative NMDAR ligands include d- and l- aspartate, homocysteine, homocysteic acid, and d-serine. NMDAR subunit composition varies substantially in different areas of the brain, and changes during development (Monyer et al., 1994; Wenzel et al., 1997). NMDAR subunits define the current amplitude and inactivation time, as well as cation selectivity and the regulation patterns, such as agonist affinity, mechano-sensitivity, Mg^2+^-sensitivity, and responsiveness to polyamines. GluN2A and GluN2B subunits contribute high channel conductance and relatively fast de-activation kinetics compared to GluN2C- and GluN2D- containing NMDAR (Traynelis et al., 2010). In addition, NMDARs containing GluN2C, GluN2D, and GluN3 subunits display low affinity for Mg^2+^ blocking the pore, making activation of such receptors independent of membrane depolarization (Monyer et al., 1994; Chatterton et al., 2002; Wrighton et al., 2008).

NMDAR sensitivity (EC_50_) to agonists is high, ranging from 0.4 to 1.7 μM for glutamate (in GluN1–GluN2D and GluN1–GluN2A receptors, respectively), and 0.1 to 2.1 μM for glycine (in GluN1–GluN2D and GluN1–GluN2A receptors, respectively) (Yamakura and Shimoji, 1999). These concentrations lie within the range that is normal in an inactive synaptic cleft. However, all types of NMDAR are extremely sensitive to the inhibition by protons (IC_50_ around 7.4 μM for most of the subunits) (Yamakura and Shimoji, 1999; Low et al., 2003; Cavara et al., 2009), and Zn^2+^ [IC_50_ of 10 nM, 1 μM, and 10 μM for the NMDAR containing GluN2A, GluN2B, and GluN2D, respectively (Gielen et al., 2009)].

NMDAR-mediated Ca^2+^ entry activates a number of intracellular signaling pathways, including Ca^2+^/calmodulin-dependent kinase (CaMK), mitogen-activated protein kinase (MAPK) [including extracellular signal-regulated kinase (ERK), Jun kinase, and p38 MAPK], and phosphoinositide 3-kinase (PI3K) (Hardingham, 2006). NMDARs regulate activity-dependent gene expression through cAMP response element binding protein (CREB) transcription factor (Hardingham et al., 2001). Other mediators downstream of NMDAR include Ras, Fyn, striatal-enriched protein tyrosine phosphatase, and nitric oxide synthase. Highly coordinated (albeit incompletely elucidated) NMDAR signaling plays critical roles in embryonic brain development and later, in neuronal plasticity, which allows the brain to respond to new experiences and changing environment (Traynelis et al., 2010).

### Unexpected Discoveries Outside of the Brain

During the past 10–20 years, NMDARs have been reported in multiple non-neuronal cell types, including hematopoietic (Bozic and Valdivielso, 2015; Hogan-Cann and Anderson, 2016), which raised a principal question of why non-excitable cells need these receptors. We admit this area of research is not very clear, sometimes even controversial, mainly due to the very low abundance of NMDAR in non-neuronal cells. Nevertheless, some progress has been achieved in the characterization of the subunit composition and currents mediated by non-neuronal NMDAR, in particular in red blood cells (RBC) (Makhro et al., 2010), platelets (Kalev-Zylinska et al., 2014), lymphocytes (Fenninger and Jefferies, 2019), and hematopoietic precursors, erythroblasts (Makhro et al., 2013; Hanggi et al., 2014, 2015) and megakaryocytes (Genever et al., 1999; Kamal et al., 2015). Information on the potential physiological role of these receptors is accumulating as well, including in erythroid cells (Makhro et al., 2013, 2016), and megakaryocytes (Hitchcock et al., 2003; Green et al., 2017; Kamal et al., 2018; Hearn et al., 2020). The subsequent sections will focus on the subunit composition, properties and the roles of NMDAR in megakaryocytic and erythroid precursors, and their mature progeny, platelets and RBCs.

## Platelet Responsiveness To Glutamate

Peripheral blood platelets store and respond to a number of regulatory molecules best known for their roles in neurotransmission, including serotonin, epinephrine, dopamine, histamine, γ-aminobutyric acid, and glutamate (Todrick et al., 1960; Ponomarev, 2018; Canobbio, 2019). In psychiatric patients, there is evidence of a crosstalk between abnormal NMDAR function in the brain and platelet responsiveness to glutamate (Berk et al., 1999). Platelets bind glutamate with similar kinetics to neurons (Almazov et al., 1988), store it in dense granules, and express AMPA, kainate, and NMDA receptors (Franconi et al., 1996, 1998; Morrell et al., 2008; Sun et al., 2009; Kalev-Zylinska et al., 2014; Green et al., 2017). Although there are variations between studies, all main types of ionotropic glutamate receptors have now been shown to be functional in platelets. Morrell et al. demonstrated that AMPA and kainate (but not NMDA) receptors amplify platelet activation by contributing Na^+^ influx to membrane depolarization, but not Ca^2+^ influx (Morrell et al., 2008; Sun et al., 2009). Franconi et al. provided the first evidence of NMDAR functionality in platelets, demonstrating that NMDARs induce Ca^2+^ influx into platelets but inhibit platelet function in the presence of adenosine diphosphate (ADP) and arachidonic acid (Franconi et al., 1996, 1998). Our own work demonstrated that NMDAR inhibitors (memantine, MK-801, and anti-GluN1 antibodies) interfere with platelet activation, aggregation and thrombus formation *ex vivo* (Kalev-Zylinska et al., 2014; Green et al., 2017). It is likely that methodological differences contributed to variable NMDAR effects between studies.

Intriguingly, in schizophrenia and bipolar disorders that are driven by deregulated NMDAR signaling, platelet Ca^2+^ levels are elevated, including in response to glutamate (Berk et al., 2000; Ruljancic et al., 2013; Harrison et al., 2019). Schizophrenia is characterized by NMDAR hypofunction in the limbic system (Coyle, 2012; Nakazawa et al., 2017), compensated by high glutamate levels and NMDAR hypersensitivity in other areas of the brain (Merritt et al., 2016). The fact that platelets from patients with schizophrenia also show glutamate hypersensitivity further argues that NMDAR functioning in platelets is similar to that in neurons (Berk et al., 2000).

Because platelets have limited protein synthesis, one would expect a similar range of glutamate receptors to be present in megakaryocytes. However, most data thus far indicate regulation of megakaryocytic differentiation by NMDAR, with little or no data on AMPA and kainate receptors (Genever et al., 1999; Hitchcock et al., 2003; Kamal et al., 2018). Nevertheless, electrophysiological recordings from freshly isolated mouse megakaryocytes support expression of functional AMPA receptors in megakaryocytes, most likely GluR2-containing and Ca^2+^-impermeable (Morrell et al., 2008).

## Glutamate and Nmdar in Megakaryocytic Cells

### Evidence for NMDAR Functionality in Megakaryocytic Cells

The first evidence that NMDARs operate as ion channels in megakaryocytes was obtained by demonstrating that [^3^H]MK-801 binds to native mouse megakaryocytes *in vivo*. Mice were injected with [^3^H]MK-801 intracardially, followed by bone marrow examination 15 min later (Genever et al., 1999). Because MK-801 is a non-competitive, use-dependent NMDAR inhibitor that can only bind within an open NMDAR pore (Traynelis et al., 2010), its labeling of megakaryocytes was consistent with the NMDAR function as an ion channel in megakaryocytic cells. Later, we showed that glutamate, NMDA, and glycine induce Ca^2+^ fluxes in Meg-01 cells, and NMDAR antagonists (MK-801, memantine, and AP5 [d-2-amino-5-phosphonopentanoate]) counteract this effect, indicating that NMDARs operate as Ca^2+^ channels in these cells (Kamal et al., 2015, 2018).

Table 1 provides a summary of the NMDAR subunit expression in megakaryocytic and erythroid cells, reported at either protein or transcript level. Unfortunately, testing for GluN proteins in hematopoietic cells has been difficult due to (a) very low abundance, (b) various protein isoforms and post-translational modifications, and (c) the lack of antibodies optimized for use in non-neuronal cells. Human megakaryocytes were first shown to express GluN1 using immunocytochemistry and Western blotting, the latter indicated that GluN1 was non-glycosylated, which may affect NMDAR distribution in the plasma membrane (Genever et al., 1999). Our group demonstrated expression of GluN1, GluN2A, and GluN2D in Meg-01, K-562, and Set-2 cells using flow cytometry and a modified Western blotting procedure that employed membrane enrichment and high-sensitivity peroxidase substrates (Kamal et al., 2015).

**Table 1:** Expression of NMDAR subunits documented in megakaryocytic and erythroid cells.

	Megakaryocytic cells^1^						Erythroid cells^2^			Mature brain cortex^3^
	Human				Mouse		Human			Mouse and human
	Normal		Leukemic		Normal		Normal – cultured			Normal
GluN subunit	Whole bone marrow	Isolated mature MKs	Cell lines	Patient-derived	Isolated mature MKs	Early cultured MKs	Proerythroblasts	Orthochromatic	Retics /RBC	Neurons
1	+; P	−	+/++; P	+	+	++; P	+	+	+; P	+++; P
2A	+	+	++; P	+	+	++	++; P	+	+; P	++; P
2B	−	−	−/+	+	−	−	−	−	−	++; P
2C	−	−	−/+	++	+	−	+; P	+++; P	++; P	+; P
2D	+	+	+++; P	+++	++	−	++; P	++; P	++; P	+; P
3A	−	−	+	++	−	−	+; P	+; P	+; P	−
3B	−	−	+++	++	++	−	+; P	++; P	++; P	−

1 Data generated mostly by RT-PCR, conventional and real-time (Genever et al., 1999; Hitchcock et al., 2003; Kamal et al., 2015, 2018).

2 Data generated by TaqMan quantitative RT-PCR, flow cytometry and immunoblotting (Makhro et al., 2013; Hanggi et al., 2014, 2015).

3 Shown by multiple techniques. The “+” symbol means expression was demonstrated; the number of “+” signs reflects the level of expression; “−” means expression was not detected. The letter “P” indicates protein expression was documented using flow cytometry or immunostaining in addition to transcript data, on which the semi-quantitative assessment was based. MK, megakaryocyte; Retics, reticulocytes; RBC, red blood cells.

The composition of NMDAR in megakaryocytes differs from that in neurons. In the brain, NMDARs are built mostly from GluN1, GluN2A, and/or GluN2B subunits, but human, native and culture-derived megakaryocytes express predominantly GluN2D, with some GluN2A and GluN1 (Table 1; Genever et al., 1999; Hitchcock et al., 2003; Kamal et al., 2015, 2018). The dominant expression of GluN2D in normal megakaryocytes will affect NMDAR functioning, however no electrophysiological recordings are available from these cells to document the effect. In other systems, GluN2D-containing NMDAR displays the following differences compared with GluN2A and GluN2B containing receptors: approximately 5-fold higher sensitivity to glutamate, 10-fold higher sensitivity to glycine, 100-fold longer deactivation time, lower conductance, lower Ca^2+^ permeability and weaker Mg^2+^ block (Paoletti et al., 2013; Wyllie et al., 2013; Hansen et al., 2018). The weak Mg^2+^ block suggests that NMDAR in megakaryocytes may not require membrane depolarization to become active; meaning the principle of “coincidence detection” may not apply. The unique functionality of the GluN2D subunit is underscored by its dominant expression in the embryonic and postnatal brain; however, the mechanism through which GluN2D subunits provide trophic effects remains incompletely understood (Watanabe et al., 1992; Akazawa et al., 1994).

For those of us working with mouse models, it is relevant to note that there are differences in the NMDAR expression patterns between human and mouse cells. In contrast to human megakaryocytes (that express only GluN1, GluN2A, and GluN2D), mouse megakaryocytes also express GluN2C and GluN3B (Table 1; Kamal et al., 2018). Small numbers of other, yet un-identified mononuclear cells in the mouse bone marrow also express NMDAR, but there is no documented expression in mouse erythroid precursors or mature RBCs (Genever et al., 1999), which differs from human cells (Table 1; Makhro et al., 2013; Hanggi et al., 2014, 2015).

In contrast to normal megakaryocytes, patient-derived leukemic megakaryoblasts and megakaryocyte leukemia cell lines (Meg-01, K-562, and Set-2) carry all possible NMDAR subunits, including GluN2B, GluN3A, and GluN3B (Table 1; Kamal et al., 2015). Meg-01 and K-562 cell lines are derived from patients with chronic myeloid leukemia in megakaryocytic and myeloid blast crisis respectively, and carry oncogenic *BCR-ABL1* gene fusion (Lozzio and Lozzio, 1975; Ogura et al., 1985). Both Meg-01 and K-562 cell lines express thrombopoietin (TPO) and erythropoietin (EPO) receptors and can be induced to differentiate into megakaryocytic (Ogura et al., 1988, Herrera et al., 1998) and erythroid cells (Andersson et al., 1979; Morle et al., 1992), thus providing experimental models of bipotential megakaryocyte-erythroid progenitors. Set-2 cell line is derived from a leukemic transformation of essential thrombocythemia and carries *JAK2* V617F mutation, an established driver in myeloproliferative neoplasms. Set-2 differentiates spontaneously into megakaryocyte-like cells (Uozumi et al., 2000). Biological characteristics of leukemic cell lines are obviously very different from normal progenitors, which we should keep in mind while interpreting cell line data.

We found that Meg-01 cells are better suited for studies of NMDAR function than K-562 and Set-2 cells, mostly because of their higher levels of NMDAR expression. Upon differentiation with phorbol-12-myristate-13-acetate (PMA), Meg-01 cells up-regulate NMDAR expression further, providing a model in which to examine NMDAR involvement in megakaryocytic differentiation (Genever et al., 1999; Kamal et al., 2018).

The role of GluN3 subunits (highly expressed in leukemic cells; Table 1) is poorly understood, including in the brain, but its functions have already been described as exquisite, peculiar, unconventional, and transformative (Kehoe et al., 2013; Perez-Otano et al., 2016; Grand et al., 2018). This is because GluN3 subunits do not require glutamate for activation (Nilsson et al., 2007). In GluN1-GluN3 receptors, glycine acts both as the sole agonist binding on GluN3, and provides feedback inhibition through GluN1. In GluN1-GluN2-GluN3 receptors, the presence of GluN3 reduces Mg^2+^ block and Ca^2+^ entry (Matsuda et al., 2002; Cavara and Hollmann, 2008).

Overall, the presence of nonconventional GluN subunits (in particular GluN2D and GluN3) in megakaryocytic cells, normal and leukemic, suggests that NMDAR generates weaker but more sustained Ca^2+^ influx, and may allow stronger modulation by glycine than glutamate, in particular in leukemic cells. There is also a possibility of regulation by metabolic factors through GluN1. This is because megakaryocytic cells express h1-1 to h1-4 GluN1 isoforms, all of type “a” (Kamal et al., 2015). GluN1a isoforms lack the N1-cassette from the N-terminal domain (encoded by exon 5) that if present, reduces inhibition by protons and zinc, and potentiation by polyamines (Traynelis et al., 1995; Yi et al., 2018). Because the bone marrow environment is intrinsically hypoxic (Spencer et al., 2014), this form of metabolic regulation warrants testing in blood progenitors.

### Evidence for Regulation of Glutamate Levels

Mouse and human megakaryocytes express a range of molecules for glutamate re-uptake, storage, and release, including VGLUT1, VGLUT2, SNARE, and the high-affinity glutamate re-uptake system, EAAT1, shown at both transcript and protein levels (Genever et al., 1999; Thompson et al., 2010). Fluorimetric measurements of glutamate concentrations in culture media suggest that human and mouse megakaryocytes, and Meg-01 cells release glutamate in a constitutive manner (Thompson et al., 2010); congruently, similar has been shown for ADP packaged together with glutamate in dense granules (Balduini et al., 2012). We do not know why megakaryocytes need glutamate sensitivity but it is a critical question to seek answers to in the future. A possible auto-regulatory loop (autocrine or paracrine) is suggested by the observation that Meg-01 cells release more glutamate upon differentiation with PMA (Thompson et al., 2010). Glutamate regulation may be independent of TPO, as NMDAR expression is maintained in megakaryocytes from c-Mpl-(TPO receptor) knockout mice (Hitchcock et al., 2003).

Unfortunately, there is no information on glutamate concentrations in the interstitial fluid of the bone marrow. In peripheral blood plasma, physiological glutamate levels are usually maintained between 20 and 100 μM (Kiessling et al., 2000), but vary widely depending on the diet (Stegink et al., 1983) and exercise (Makhro et al., 2016). In the interstitial fluids, concentrations of glutamate have been observed to be as low as 0.5 μM in the masseter muscle of myofascial temporomandibular joint (Castrillon et al., 2010), to as high as blood plasma levels in the vastus lateralis muscle of the lower limb (Gerdle et al., 2016). There is no experimental data on how plasma/interstitial glutamate levels impact endogenous NMDAR in blood/progenitor cells. Paracrine NMDAR activation in neuron-like fashion appears more likely in tissues with low, steady state glutamate concentrations. On the other hand, conditional NMDAR activation *via* local pH changes would be more likely in tissues with higher, plasma-like glutamate concentrations. Different types of NMDAR subunits will also affect cellular sensitivity to glutamate, and modulate the receptor response (as described in section Evidence for NMDAR Functionality in Megakaryocytic Cells).

### NMDAR Effects on Megakaryocytic Differentiation

**Figure 1:**
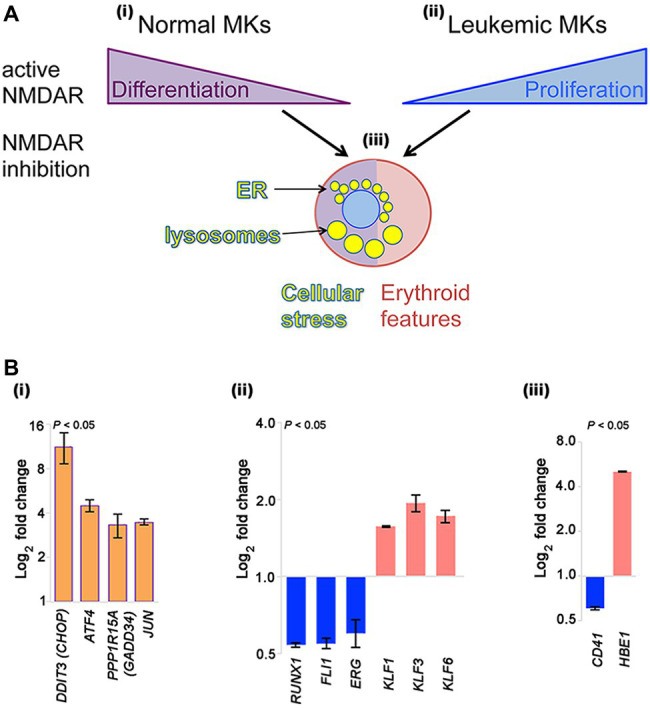
NMDAR effects in normal megakaryocytes and leukemic Meg-01 cells. **(A)** Schematic indicating that in normal megakaryocytes NMDAR activity supports differentiation, in particular proplatelet formation **(i)**. In contrast, in leukemic cell lines NMDARs increase cell proliferation **(ii)**. In both normal and leukemic cells, NMDAR inhibition induces cellular stress response associated with endoplasmic reticulum (ER) dilatation and accumulation of lysosomes **(iii)**. Red shade in a cell reflects features of erythroid differentiation. **(B)** Experimental data showing that CRISPR-Cas9-mediated deletion of *GRIN1* in Meg-01 cells increased expression of ER stress markers (*DDIT3/CHOP*, *ATF4*, *PPP1R15A*/*GADD34*, and *JUN*; **Bi**; orange bars), associated with decreased expression of megakaryocytic transcription factors (*RUNX1*, *FLI1*, *ERG*; **Bii**) and megakaryocytic maturation marker, CD41 **(Biii)** (blue bars). Instead, expression of erythroid transcription factors (*KLF1*, *KLF3*, *KLF6*; **Bii**) and embryonic hemoglobin (*HBE1*; **Biii**) was increased (red bars). Transcript levels were determined by real-time RT-PCR **(Bi)** and Clariom S microarrays **(Bii–iii)**, as described (Hearn et al., 2020). Statistical significance is shown (*p* < 0.05 for all markers versus unmodified Meg-01 cells set at 1.0, tested by one-way ANOVA with Dunnett post-hoc. MK, megakaryocyte.

NMDAR channel blockers (memantine and MK-801) induce two types of apparently opposing effects in cultured megakaryocytic cells: inhibition of differentiation in normal megakaryocytes, but induction of differentiation in megakaryocytic leukemia cell lines (Figure 1Ai–ii).

When human megakaryocytes are grown from CD34-positive umbilical cord stem cells, the addition of MK-801 inhibits acquisition of megakaryocytic markers (CD61, CD41a, and CD42a), nuclear ploidy and proplatelet formation; however, progenitor proliferation is unaffected (Hitchcock et al., 2003). Similar effects are seen in cultures of mouse hematopoietic progenitors, and in the native bone marrow milieu of mouse bone marrow explants. MK-801 inhibits actin reorganization in mature mouse megakaryocytes, suggesting that NMDAR-mediated Ca^2+^ influx is required for the cytoskeletal remodeling that underlies proplatelet formation (Kamal et al., 2018). This process may be similar to dendritic spine formation arising in response to neuronal NMDAR firing (Furuyashiki et al., 2002). NMDAR links with cytoskeletal elements through post-synaptic density (PSD) proteins such as PSD-95 and Yotiao; both of which are expressed in megakaryocytes, suggesting similar interactions may be possible in hematopoietic cells (Hitchcock et al., 2003).

In contrast to normal megakaryocytes that utilize NMDAR function to assist differentiation, leukemic cell lines (Meg-01, K-562, and Set-2) appear to divert NMDAR activity to increase proliferation (Figure 1Ai–ii). In the presence of NMDAR blockers (memantine and MK-801) Meg-01 cells undergo atypical differentiation and accumulate prominent cytoplasmic vacuoles (Figure 1Aiii; Kamal et al., 2015, 2018). The opposing NMDAR effects on cellular phenotype between normal and leukemic cells suggest divergence of NMDAR pathways during leukemogenesis to increase cell proliferation.

To get more insights into the mechanism of this divergence, we recently created a model of NMDAR hypofunction in Meg-01 cells using CRISPR-Cas9 mediated knockout of the *GRIN1* gene that encodes the obligate, GluN1 subunit of the NMDAR (Hearn et al., 2020). We found that *GRIN1* deletion caused marked changes in the intracellular Ca^2+^ homeostasis, including higher cytosolic Ca^2+^ levels at baseline but lower ER Ca^2+^ release after activation. Deregulated Ca^2+^ handling led to endoplasmic reticulum (ER) stress and induced autophagy. Prominent cytoplasmic vacuoles accumulated in Meg-01-*GRIN1* ^−/−^ cells and were found to represent dilated ER and lysosomal organelles (Figure 1Aiii). Microarray analysis revealed that Meg-01-*GRIN1* ^−/−^ cells had deregulated expression of transcripts involved in Ca^2+^ metabolism, together with a shift in the pattern of hematopoietic transcription factors toward erythropoiesis (Figure 1Bi–ii). In keeping with the pro-erythroid pattern of transcription factors, Meg-01-*GRIN1* ^−/−^ cells displayed features of erythroid differentiation (Figure 1Biii). Our data provide the first evidence that NMDARs comprise an integral component of the Ca^2+^ toolkit in megakaryocytic cells, and argue that intracellular Ca^2+^ homeostasis may be more important than currently recognized for balancing megakaryocytic with erythroid differentiation at the level of a common progenitor (Hearn et al., 2020).

In support of our findings, Kinney et al. provided computational evidence of NMDAR involvement in erythropoiesis (Kinney et al., 2019). The authors analyzed 164 publicly available erythroid microarray datasets using an enhanced CellNet bioinformatics algorithm to delineate key transitional states of erythroid differentiation at high resolution. This approach identified a role for signaling through epidermal growth factor receptor Erb-B2 receptor tyrosine kinase 4 (ErbB4) in erythroid differentiation, which was further validated experimentally in zebrafish, mouse and human models. The authors linked ErbB4 with NMDAR signaling by finding increased levels of *GRIN3B* transcripts, coding for GluN3B, in the reticulocyte gene cluster. A similar link between ErbB4 and NMDAR is well-documented in neurons, where ErbB4 and its neuregulin ligands stabilize synaptic NMDAR (Li et al., 2007). In fact, altered neuregulin 1–ErbB4 signaling is a well-established mechanism of NMDAR hypofunction in schizophrenia (Hahn et al., 2006). Encouragingly, we also found that in Meg-01-*GRIN1* ^−/−^ cells, transcripts for neuregulin 1 and ErbB receptor feedback inhibitor 1 were up-regulated (1.98- and 2.05-fold, respectively), implying the ErbB4-NMDAR link is maintained during megakaryocytic-erythroid differentiation (Hearn et al., 2020).

The interrogation of publicly available transcriptomic data obtained from human megakaryocyte-erythroid progenitors at a single cell level demonstrated the presence of *GRINA* transcripts (encoding NMDAR-associated protein 1, known to be expressed at relatively high levels) and a scatter of low signals for *GRIN1*, *GRIN2A*, *GRIN2C*, and *GRIN2D* (Lu et al., 2018). Deep sequencing in that study was performed with approximately 3 million reads per cell, which captured approximately 6,000 of the most highly expressed transcripts in a cell, which may explain why *GRIN* transcripts were detected at very low levels.

## Nmdar Functionality in Erythroid Cells and in the Circulating Rbcs

RBCs sense plasma glutamate levels through the NMDAR. Using radiolabeled antagonist ([^3^H]MK-801) binding assay, basal activity of NMDARs in RBCs suspended in plasma was shown (Makhro et al., 2013). Supplementation of glutamate to plasma caused further activation of the receptors (Hanggi et al., 2014). Other findings suggest that the shear of flowing blood may also activate NMDAR in RBCs of patients with sickle cell disease (Hanggi et al., 2014, 2015).

### NMDAR in Erythroid Precursor Cells

The abundance of NMDARs is particularly high in erythroid precursors and the UT-7/EPO cell line (Makhro et al., 2013; Hanggi et al., 2015). The receptor density decreases from hundreds of thousands per cell in proerythroblasts and erythroblasts cultured from peripheral blood-derived CD34-positive progenitors, to 35 in young human RBCs, and five in mature and senescent RBCs from healthy people (Makhro et al., 2013; Hanggi et al., 2014). In UT-7/EPO cell line 350,000 NMDARs were detected per cell (Makhro et al., 2010). UT-7/EPO is a subclone of a UT-7 megakaryoblastic leukemia cell line that was maintained in the presence of EPO for more than 6 months to increase erythroid differentiation (Komatsu et al., 1993). In keeping with the NMDAR expression data, the density of currents produced by the NMDAR decreased during differentiation from proerythroblastic to the orthochromatic stage of erythroid progenitors (Hanggi et al., 2015).

Similar to megakaryocytes, the pattern of GluN subunits evolves during erythroid differentiation. Except for GluN2B, all other types of NMDAR subunits have been detected in erythroid cells at either mRNA or protein levels, or both (Table 1). During early stages, (proerythroblasts and basophilic erythroblasts) higher levels of GluN2A and GluN2D were shown along with lower levels of GluN3A, GluN3B, and GluN1 (Makhro et al., 2013; Hanggi et al., 2014, 2015). Orthochromatic erythroblasts switched from GluN2A-containing receptors to those predominantly containing GluN2C (Hanggi et al., 2015). As a result, high amplitude, fast, inactivating currents, mediated by the receptor at early stages of erythroid differentiation were replaced by currents of lesser amplitude but longer duration (Hanggi et al., 2015). This change in receptor subunit composition and its function gave rise to a switch in signal transmitted by the NMDAR, and probably, to the alteration in sensitivity to the physiological stimuli. Whereas GluN2A-containing receptors contributed to the modulation of the transmembrane potential, GluN2C/GluN2D-NMDAR mediated Ca^2+^ entry through the channels that remained open for a longer time (Hanggi et al., 2015). Mg^2+^ block is not supposed to control the NMDAR activity in RBCs due to the low transmembrane potential (about −10 mV) and the presence of GluN2C, GluN2D, and GluN3 subunits (Monyer et al., 1994; Wrighton et al., 2008).

Hyperactivation of the NMDAR by repeated stimulation resulted in the channel inactivation and did not affect viability of erythroid precursor cells. However, exposure of erythroid progenitors to the NMDAR channel blockers (MK-801 and memantine) triggered vacuolization and apoptosis, with maximal cell death observed at the early differentiation stages (Hanggi et al., 2014, 2015). This observation is in line with the earlier findings by Miller and Cheung on the importance of Ca^2+^ signaling for EPO-driven effects in precursor cells (Miller and Cheung, 1994; Tong et al., 2008).

### NMDAR Function and Physiological Significance in the Circulating RBCs

Relatively low numbers of active NMDAR copies are retained by the circulating RBCs. Young RBCs of healthy humans carry 35 NMDARs per cell on average, whereas mature and senescent cells contain about five receptor copies per cell (Makhro et al., 2013; Hanggi et al., 2014). The NMDAR abundance is 3–4-fold higher in RBCs from patients with sickle cell disease (Hanggi et al., 2014). Activation of NMDAR by exposing the cells to the saturating concentrations of agonists (NMDA and glycine, 300 μM each) results in an acute, transient increase in the intracellular free Ca^2+^ (Makhro et al., 2013). There is striking inter-cellular heterogeneity in responses of the cells to the NMDAR agonists, including changes in transmembrane currents and Ca^2+^ uptake shown with a fluorescent dye, as well as the level of dehydration and echinocyte formation. These differences cannot be explained solely by the differences in RBC age. Whereas some cells are insensitive to the stimulation, others show a clear response to the NMDAR agonists suggesting inter-cellular heterogeneity in NMDAR numbers/distribution. Along with the changes in RBC volume and density of Ca^2+^ uptake following the NMDAR stimulation, we observed regulation of nitric oxide production in RBCs by the nitric oxide synthase and modulation of the redox state (Makhro et al., 2010).

Physiological responses to the changes in NMDAR activity in the circulating RBCs include regulation of hemoglobin oxygen affinity, cell rheology, and most likely, longevity. Pathophysiological downstream effects associated with NMDAR hyperactivation were revealed *ex vivo* for RBCs of patients with sickle cell disease. These included Ca^2+^ overload, dehydration, and increase in cell density, and oxidative stress (Hanggi et al., 2014). There are no reports of abnormal RBC counts in patients with Alzheimer’s disease taking memantine to protect the brain from glutamatergic excitotoxicity (Kavirajan, 2009). One pilot clinical trial was performed at the University Hospital Zurich, in which patients with sickle cell disease received memantine over a year (Makhro et al., 2020; trial identifier NCT02615847), and the other is currently ongoing (trial identifier NCT03247218). These trials provide an opportunity to explore long-term effects of NMDAR inhibition on RBC and platelet production, and cell properties in humans.

## Conclusions and Future Directions

In summary, megakaryocytic and erythroid precursors carry nonconventional NMDAR subunits, therefore NMDAR activity during hematopoiesis may be unique and should be tested. NMDARs regulate megakaryocytic and erythroid differentiation *ex vivo*, and balance both fates during differentiation of Meg-01 cells, suggesting NMDAR role at the level of a bipotential megakaryocyte-erythroid progenitor. NMDAR effects in hematopoietic cells are mediated by Ca^2+^ influx, which in early megakaryoblasts affects transcriptional program of differentiation, and in mature megakaryocytes induces cytoskeletal rearrangements required for proplatelet formation. In contrast to normal progenitors, leukemic cell lines re-direct NMDAR signaling to increase proliferation. The shift in the dominant NMDAR effect in leukemic cells may be at least partially related to different GluN subunits these cells express, which may offer therapeutic opportunities.

In keeping with the proliferative NMDAR effects in leukemic cells, other prominent groups found that GluN2B-containing NMDAR promotes growth of pancreatic tumors (Li and Hanahan, 2013; Li et al., 2018), and enable brain metastases by breast cancer (Zeng et al., 2019). The following molecules acting downstream of NMDAR were shown to assist cancer spread in these studies: CaMKII, MAPK, guanylate-kinase-associated protein, heat shock factor 1, and fragile X mental retardation protein (Li and Hanahan, 2013; Li et al., 2018); we should examine similar pathways in leukemic cells as they may provide novel therapeutic targets.

In cultured human proerythroblasts, GluN2A-containing NMDAR provides depolarization, and inward Ca^2+^ current of high amplitude and fast inactivation kinetics. The presence of active NMDAR supports survival of early erythroid progenitors, which likely contributes to the EPO-driven signaling; however, this link is still to be demonstrated. Expression of GluN2C and GluN2D in the late erythroid precursors coincides with the onset of hemoglobinization. Thus, the NMDAR role in iron uptake warrants investigation. In mature RBCs, NMDAR regulates basal intracellular Ca^2+^ levels, contributing toward the regulation of cell volume, density, redox balance, and nitric oxide production by RBCs, which most likely contributes to the regulation of RBC longevity and oxygen carrying capacity.

NMDAR signaling can be modulated using small molecules. Memantine is an approved drug for neurological patients and could be repurposed against certain hematological disorders, such as sickle cell disease, megakaryocytic cancers and thrombosis. Preclinical studies have already advanced to stage I clinical trials in sickle cell disease, but a lot more needs to be done to determine if NMDAR modulation could be useful in patients with certain myeloid blood cancers or thrombotic disease. Neurological side effects may limit the use of memantine in hematological patients; therefore, alternative strategies may need to be considered. These include subunit-specific NMDAR inhibitors, compounds that do not cross the blood-brain-barrier, and drugs that target pathways downstream, or glutamate release upstream of NMDAR.

Considering that the NMDAR role in megakaryocytic cells was first reported in Genever et al. (1999), the progress in this field may be viewed as relatively modest. However, we have reached a state of acceptance that NMDARs provide meaningful biological effects in hematopoietic cells. The field is attracting renewed attention. We await results from the first, stage I clinical trial in patients with sickle cell disease, primarily to establish safety of memantine outside of neurological indications. Further progress into NMDAR role in human leukemia and thrombosis will require studies in more advanced *ex vivo* and *in vivo* models. In addition, the overall principle and purpose of peripheral glutamate signaling needs to be determined. We, thus, invite collaborative approaches engaging experts from multiple disciplines to join us forming an interest group focusing on peripheral glutamate signaling.

## Author Contributions

MK-Z, AM and AB wrote the paper. JH contributed research data. All authors approved the final version for submission.

### Conflict of Interest

The authors declare that the research was conducted in the absence of any commercial or financial relationships that could be construed as a potential conflict of interest.

## Abbreviations

ADP, adenosine diphosphate; AMPA, α-amino-3-hydroxy-5-methyl-4-isoxazolepropionic acid; AP5, D-2-amino-5-phosphonopentanoate; CaMK, Ca^2+^/calmodulin-dependent kinase; CREB, cAMP response element binding protein; EAAT, excitatory amino acid transporters; EC_50_, the concentration of an agonist that gives half-maximal response; EPO, erythropoietin; ER, endoplasmic reticulum; ErbB4, epidermal growth factor receptor Erb-B2 receptor tyrosine kinase 4; ERK, extracellular signal-regulated kinase; IC_50_, the concentration of an inhibitor where the response (or binding) is reduced by half; MAPK, mitogen-activated protein kinase; MEP, megakaryocyte-erythroid progenitor; MK, megakaryocyte; NMDAR, *N*-methyl-d-aspartate receptor; PI3-K, phosphoinositide 3-kinase; PMA, phorbol 12-myristate 13-acetate; PSD, post-synaptic density; RBC, red blood cell; SNARE, soluble *N*-ethyl maleimide-sensitive factor attachment protein receptor; TPO, thrombopoietin; VGLUT, vesicular glutamate transporter.
